# Supplementary figures and images for: Psychological stress disrupts intestinal epithelial cell function and mucosal integrity through microbe and host-directed processes
Source: Gut Microbes. 2022 Feb 20;14(1):2035661. doi: 10.1080/19490976.2022.2035661 (PMC8865257; doi:10.1080/19490976.2022.2035661)

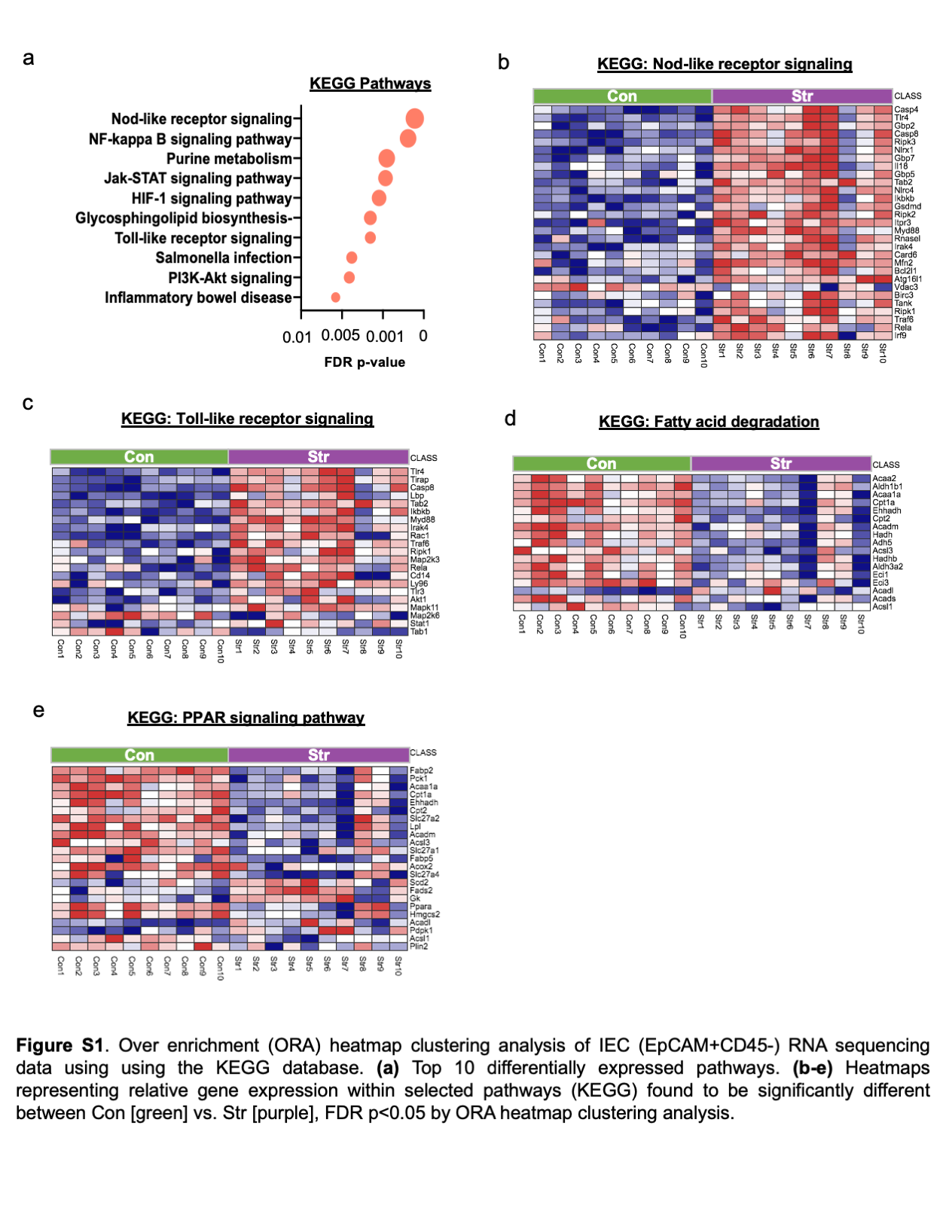

Supplement: Supplemental Material [file KGMI_A_2035661_SM5741.zip › supplementary/FigureS1.tiff]

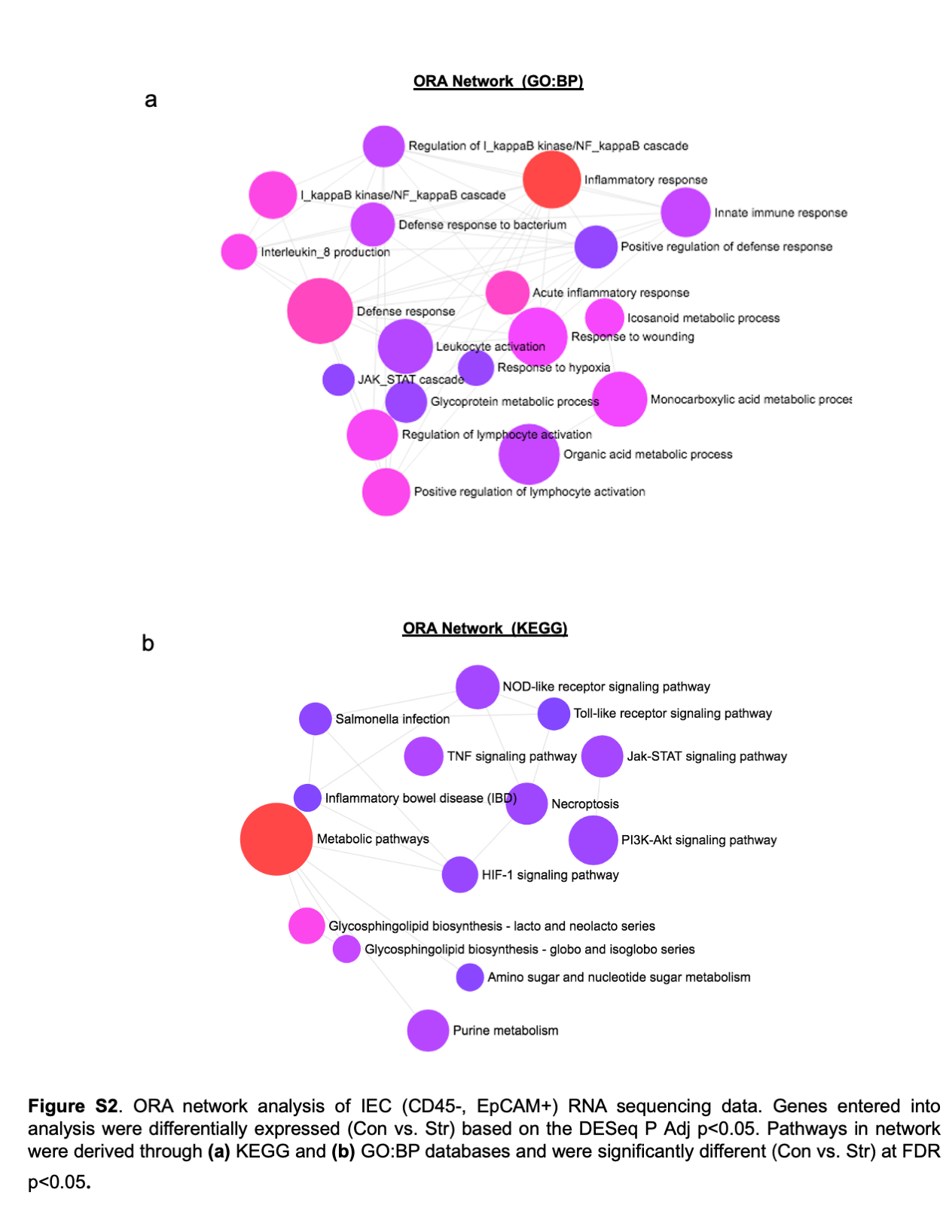

Supplement: Supplemental Material [file KGMI_A_2035661_SM5741.zip › supplementary/FigureS2.tiff]

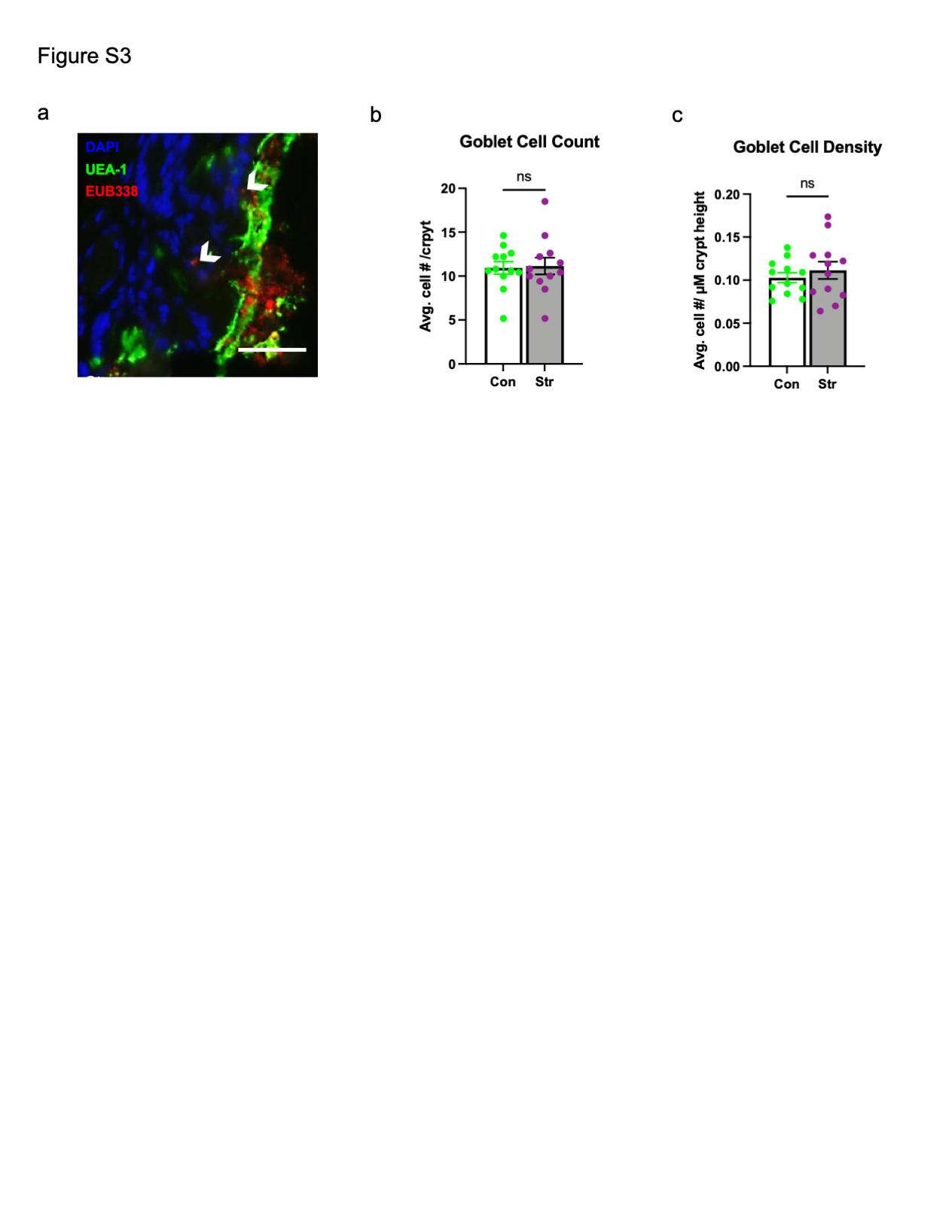

Supplement: Supplemental Material [file KGMI_A_2035661_SM5741.zip › supplementary/FigureS3.tiff]

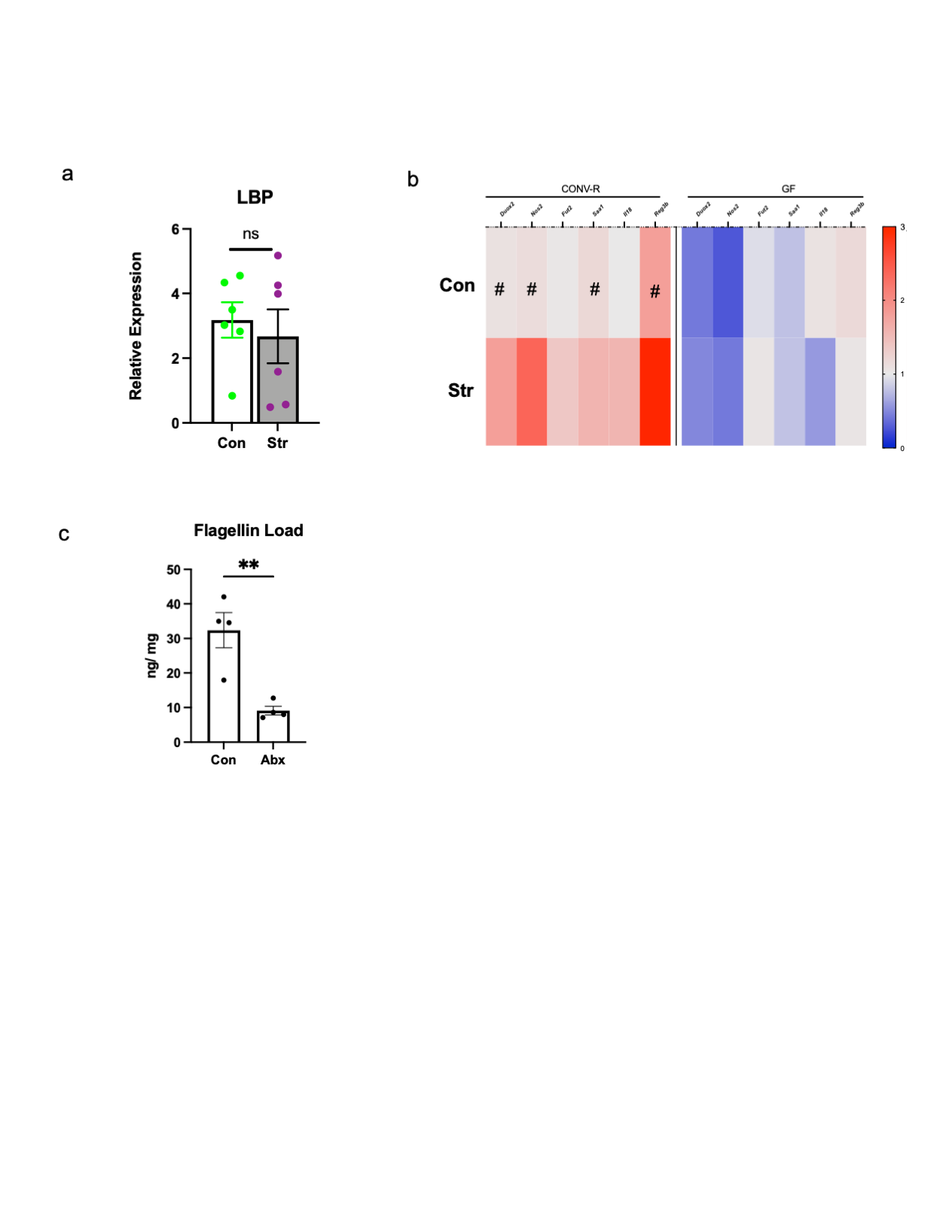

Supplement: Supplemental Material [file KGMI_A_2035661_SM5741.zip › supplementary/FigureS4_New.tiff]

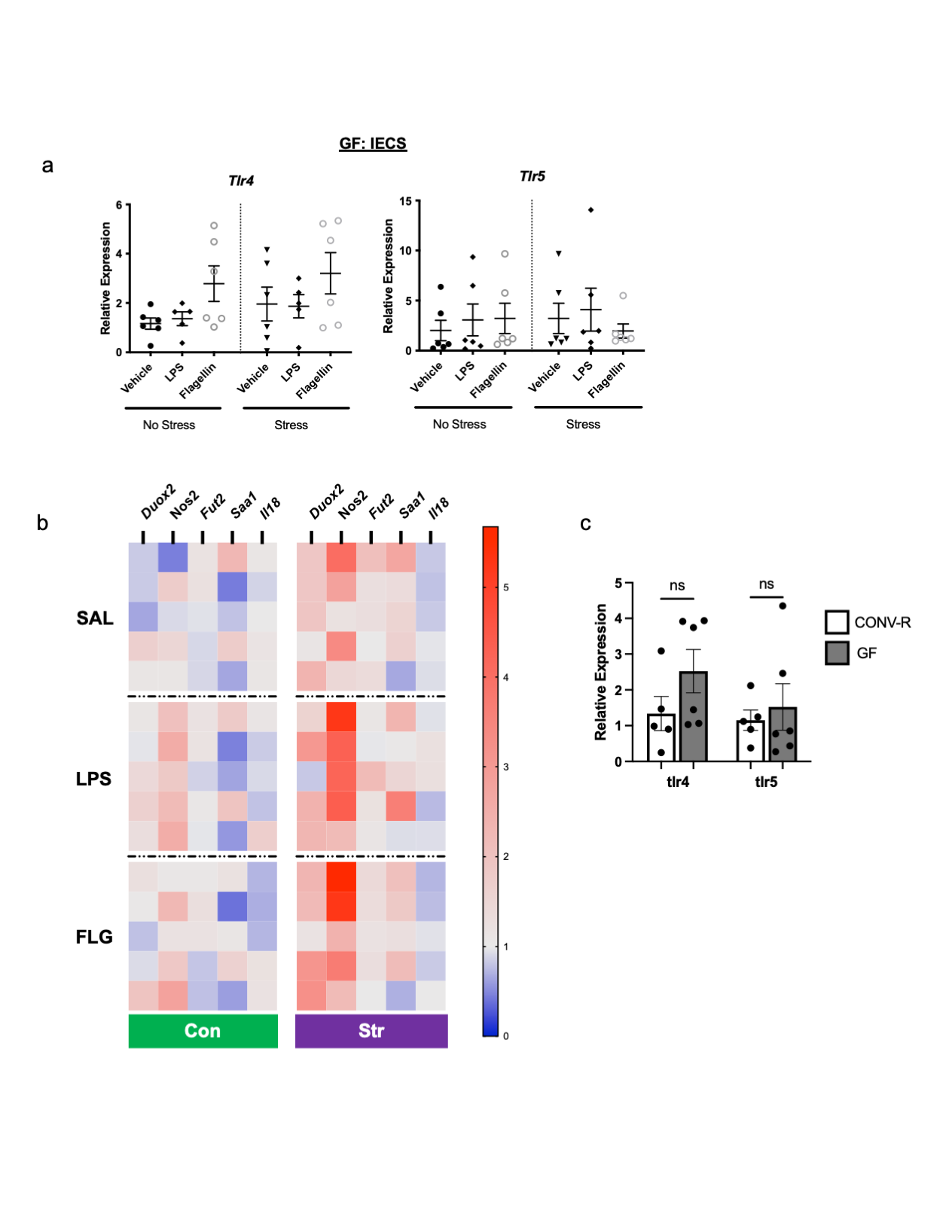

Supplement: Supplemental Material [file KGMI_A_2035661_SM5741.zip › supplementary/FigureS5.tiff]
